# Predicting the risk of early bleeding following endoscopic variceal ligation in cirrhotic patients with computed tomography

**DOI:** 10.1186/s12876-023-03038-1

**Published:** 2023-11-24

**Authors:** Hwaseong Ryu, Tae Un Kim, Ki Tae Yoon, Young Mi Hong

**Affiliations:** 1 Department of Radiology, Pusan National University College of Medicine, Pusan National University Yangsan Hospital, Yangsan, Republic of Korea; 2 Department of Internal Medicine, Pusan National University College of Medicine, Pusan National University Yangsan Hospital, Yangsan, Republic of Korea

**Keywords:** Esophageal varices, Endoscopic variceal ligation, Computed tomography, Liver cirrhosis

## Abstract

**Background:**

Life-threatening bleeding following endoscopic variceal ligation (EVL) in patients with cirrhosis rarely can occur. The present study aimed to evaluate the performance of computed tomography (CT) in predicting the risk of early bleeding following EVL in cirrhotic patients.

**Methods:**

We retrospectively investigated 285 cirrhotic patients who had undergone EVL. EVL was performed for prophylaxis or acute variceal bleeding. The patients were classified into 2 groups: early bleeding (< 14 days after EVL) and non-early bleeding. We compared baseline characteristics including CT findings between the patient groups.

**Results:**

Among the 285 patients who underwent EVL treatment, 19 patients (6.7%) experienced early bleeding. On average, these bleeding occurred 9.3 ± 3.5 days after the EVL, with a range of 3 to 13 days. Patients who experience early bleeding had a higher six-week bleeding-related mortality rate compared to those in the non-early bleeding group (31.6% vs. 10.2%; *p* = 0.014). There was a correlation between the grade of esophageal varix observed during endoscopy and the diameter of esophageal varix observed on CT (*p* < 0.001). The diameter of esophageal varix on CT was identified as the only significant predictive factor for early bleeding (*p* = 0.005).

**Conclusion:**

A larger esophageal varix diameter observed on CT is associated with an increased risk of early bleeding after EVL treatment. Early identification of this high-risk group can provide a change of treatment strategies to improve patient outcomes.

## Background

Gastroesophageal varices is one of the most common complications of advanced liver cirrhosis. Of Child-Pugh A patients, 45% have gastroesophageal varices and the prevalence increases up to 72% of Child-Pugh B/C patients [1]. Variceal bleeding, causative of 70% of all upper gastrointestinal bleeding, has an occurrence rate of 5–15% per year in patients with cirrhosis [2–4]. It remains one of the most severe and immediate life-threatening complications and mortality from esophageal variceal bleeding is as high as 20% [5]. The risk of variceal bleeding is related to variceal size and other reported predictors are decompensated cirrhosis (Child-Pugh B/C) and the endoscopic presence of red wale marks on the varices [6].

Current guidelines recommend non-selective beta-blockers or endoscopic variceal ligation (EVL) for primary prophylaxis of esophageal variceal bleeding. When acute variceal bleeding is confirmed by endoscopy, EVL should be performed. Despite treatment with vasoactive drugs plus EVL and prophylactic antibiotics, up to 10–15% of patients with acute variceal bleeding have persistent or early rebleeding [7–9].

Early bleeding after EVL is reported mainly due to early slippage of rubber bands and is fatal in some cases. Several previous studies have suggested the possible predictive factors for early bleeding after EVL: previous variceal bleeding, peptic esophagitis, low albumin, high D-dimer, presence of ascites, high aspartate aminotransferase to platelet ratio index (APRI) score, low prothrombin index, number of varices, extent of varices, and number of rubber bands [10–13]. Since these studies included heterogenous populations and the risk factors were not well validated, until now, there was no general consensus about the predictive factors and strategies to prevent early bleeding.

Patients with cirrhosis occasionally undergo multiphase computed tomography (CT) scans to screen for hepatocellular carcinoma (HCC). The evaluation of portosystemic collateral vessels, including gastroesophageal varices, using CT scans is possible. Several previous studies have evaluated the correlation between CT findings and endoscopy and have demonstrated that it was comparable to endoscopy in detecting varices [14–16]. In addition, other studies have shown that a CT scan is much better tolerated and more cost-effective than endoscopy [14, 17, 18]. Currently, however, studies have not examined the utility of CT scan in predicting the risk of early bleeding after EVL. The present study aimed to evaluate CT performance in predicting the risk of early bleeding following EVL, in patients with cirrhosis.

## Patients and methods

### Patients

**Fig. 1:**
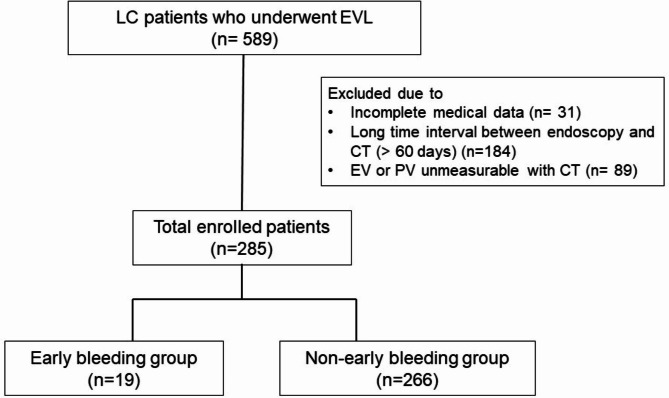
Flow diagram of patient enrollment

Consecutive patients who had undergone EVL in Pusan National University Yangsan Hospital from January 2009 to December 2018 were retrospectively analyzed. EVL was performed for prophylaxis or acute bleeding of esophageal varices (EV). All patients received vasoactive drugs (terlipressin/somatostatin) and intravenous broad-spectrum antibiotics according to the guidelines [6]. Food intake after EVL was allowed at the discretion of the physician. The exclusion criteria included patients with incomplete medical records, those with a long time interval between endoscopy and CT (> 60 days), and those with unmeasurable EV or portal veins on the CT scan (Fig. 1). Laboratory results for all patients were retrieved on the day of EVL procedure.

According to the occurrence of early bleeding after EVL (bleeding occurring between 24 h and 14 days after the procedure), the patients were classified into 2 groups. Bleeding after EVL was defined according to the Baveno criteria as follows: (1) recurrent hematemesis, and/or melena, and/or bloody fluid drained by the nasogastric tube or (2) a decrease in hemoglobin by at least 2 g/dL, or a transfusion of more than 2 units of packed RBCs needed within 24 h, or hypovolemic instability [19]. Proton pump inhibitors (PPIs) were not administered after EVL [20, 21]. Bleeding-related death was defined as death within 6 weeks of the index bleeding [22]. The present study was conducted in accordance to the ethical guidelines of the Declaration of Helsinki and was approved by the Institutional Review Boards (IRB) of Pusan National University Yangsan Hospital. Requirement for informed consent was waived after review of IRB of Pusan National University Yangsan Hospital because it was practically impossible and this study was of retrospective design.

### Endoscopic procedure for EVL

Upper endoscopy was performed using Olympus GIFQ 260 or Pentax EG 2940 scope. The grade of EV was classified as small straight (F1); enlarged tortuous, occupying less than one third of the esophageal lumen (F2); or large sized, coil-shaped that occupy more than one-third of the esophageal lumen (F3) [23]. EVL was performed using a multiband ligator (6 shooter Saeed Multi-band ligator, Cook Medical Endoscopy, Limerick, Ireland).

### CT measurements

All patients underwent abdominal CT imaging including portal venous phase image. Different CT systems (Somatom Definition Flash, Somatom Definition AS+, or Somatom Sensation 16, Siemens Medical Systems, Erlangen, Germany; and Discovery 750HD, Revolution, General Electric Medical Systems, Milwaukee, WI, USA) were used with slice thickness of 5 mm, and a 3 mm reconstruction interval. CT images were obtained with settings of 100 or 120 kVp, and mAs was set by automatic exposure control for each patient. Portal venous image was obtained 60 s after contrast media injection during suspended inspiration. The CT images were evaluated with consensus by two abdominal radiologists (T.U.K with 12 years of experience, and H.R with 6 years of experience) who were blinded to the patient’s medical history, and laboratory and endoscopic findings. Maximum short axis diameter of the largest esophageal varix and maximum main portal vein diameter of each patient were recorded. Maximum main portal vein diameters were measured at a point at least 1 cm distal to the confluence of the splenic and superior mesenteric veins and at least 1 cm proximal to the first branch of the main portal vein on axial portal venous phase images [24].

### Statistical analysis

Continuous variables between the early and non-early bleeding groups were analyzed using a Student`s t-test. The Chi-square test or Fisher`s exact test was used to investigate the association between categorical factors and early bleeding. Differences between groups of continuous variables that were non-parametrically distributed were assessed using Kruskal–Wallis tests. All variables selected in univariate analysis (*p* < 0.20) were included in the multivariate model. In the multivariate analysis, logistic regression models were used to investigate the independent risk factors associated with early bleeding after EVL. Receiver operator characteristics (ROC) curves were used to evaluate the optimal cut-off value for EV diameter on CT. Statistical analysis was performed using SPSS for Windows (version 21; SPSS Inc., Chicago, IL, USA). All *p*-values < 0.05 were considered statistically significant.

## Results

### Baseline characteristics of the enrolled patients

A total of 285 patients who underwent EVL were included in our study. The baseline characteristics of the enrolled patients are shown in Table 1. Our study included 232 (81.4%) males and 53 (18.6%) females. The mean age was 60.5 ± 10.7 years. There were 89 (31.2%), 144 (50.5%), and 52 (18.3%) patients with Child-Pugh classification of A, B, and C, respectively. In 16 (5.6%), 92 (32.3%), and 177 (62.1%) patients, the EV grades were F1, F2, and F3, respectively. Eighty-three (29.1%) patients had a concomitant gastric varices (GV) and 33 (11.6%) patients had a previous history of variceal bleeding. Twenty six (9.1%) patients had portal vein thrombosis, and 122 (42.8%) patients had HCC. Model for end-stage liver disease (MELD) score was 13.7 ± 5.6 and prothrombin time (PT) was 16.3 ± 3.1 sec.

**Table 1:** Baseline clinical characteristics of the enrolled patients

	N = 285
Age (yr)	60.5 ± 10.7
Gender (Male)	232 (81.4%)
Etiology	
Hepatitis virus B	115 (40.4%)
Hepatitis virus C	32 (11.2%)
Alcohol	106 (37.2%)
Others	32 (11.2%)
Child-Pugh score	
A/B/C	89 (31.2%)/ 144 (50.5%) / 52(18.3%)
MELD	13.7 ± 5.6
Presence of Ascites	170 (59.7%)
Presence of Hepatic encephalopathy	55 (19.3%)
Esophageal varices grade	
F1/F2/F3	16 (5.6%)/ 92 (32.3%) / 177 (62.1%)
Presence of GV	83 (29.1%)
Previous history of variceal bleeding	33 (11.6%)
Presence of PVT	26 (9.1%)
Presence of HCC	122 (42.8%)
Hemoglobin, g/dL	9.25 ± 2.09
Platelet, x10^6^/L	94428.1 ± 52882.5
Total bilirubin, mg/dL	3.1 ± 4.7
Albumin, g/dL	3.1 ± 0.5
Prothrombin time, secs	16.3 ± 3.1
Na, mmol/L	135.9 ± 5.1
Previous ß-blockers treatment	65 (22.8%)

Data are presented as number (%) or mean ± SD.

GV, gastric varices; HCC, hepatocellular carcinoma; MELD, model for end-stage liver disease; PVT, portal vein thrombosis

### Comparison of baseline characteristics between early bleeding and non-early bleeding groups

Among the 285 patients treated with EVL, 19 patients (6.7%) developed early bleeding. Early bleeding occurred with a mean day of 9.3 ± 3.5 days (range: 3–13 days). Six weeks bleeding-related death rate was 11.6% (33/285).

The characteristics of both the early and non-early bleeding groups are presented in Table 2. There was no difference between groups regarding various factors except six-week bleeding-related mortality rate. The severity of the liver disease was similar in both groups [Child Pugh class score (*p* = 0.313) and MELD (*p* = 0.223)]. Previous history of variceal bleeding (*p* = 0.553)], indication of EVL (*p* = 0.835), presence of ascites (*p* = 0.197), the presence of portal vein thrombosis (*p* = 0.943), PT (*p* = 0.157) and total bilirubin (*p* = 0.050) were not significantly different between groups. Six-week bleeding-related mortality rate was significantly higher in early bleeding group than in the non-early bleeding group (31.6% vs. 10.2%; *p* = 0.014).

**Table 2:** Comparison of baseline characteristics between the patient groups

Variables	Non-early bleeding (N = 266)	Early bleeding (N = 19)	P value
Age	60.65 ± 10.695	58.89 ± 11.396	0.492
Gender (Male)	217 (81.6%)	15 (78.9%)	0.776
Etiology (HBV/HCV/Alcohol/Others)	109(41.1%)/30(11.3%) /99(37.2%)/28(10.5%)	6(31.6%)/2(10.5%) /7(36.8%)/4(21.1%)	0.563
Previous history of variceal bleeding	30 (11.3%)	3 (15.8%)	0.553
Indication of EVL			0.835
prophylactic	47 (17.7%)	3 (15.8%)	
emergency	219 (82.3%)	16 (84.2%)	
Presence of PVT	24 (9.0%)	2 (10.5%)	0.943
Presence of HCC	114 (42.9%)	8 (42.1%)	0.962
Child-Pugh score			0.313
A	84 (31.6%)	5 (26.3%)	
B	136 (51.1%)	8 (42.1%)	
C	46 (17.3%)	6 (31.6%)	
Presence of Ascites	156 (58.6%)	14 (73.7%)	0.197
Presence of Hepatic encephalopathy	50 (18.8%)	5 (26.3%)	0.703
Hemoglobin, g/dL	9.25 ± 2.107	9.21 ± 1.913	0.935
Platelet, x10^6^/L	94936.09 ± 53070.05	87315.79 ± 51012.04	0.545
Prothrombin time, sec	16.20 ± 3.11	17.26 ± 3.20	0.157
Total bilirubin, mg/dL	2.97 ± 4.20	5.17 ± 9.36	0.050
Albumin, g/dL	3.08± 0.53	3.02 ± 0.54	0.629
Na, mmol/L	135.93 ± 5.14	135.63 ± 5.23	0.808
Child Pugh score	7.74 ± 2.02	8.52 ± 2.71	0.233
MELD	13.63 ± 5.55	15.26 ± 6.57	0.223
6weeks bleeding-related mortality	27(10.2%)	6(31.6%)	0.014

Data are presented as number (%) or mean ± SD.

EVL, esophageal variceal ligation; HCC, hepatocellular carcinoma; MELD, model for end-stage liver disease; PVT, portal vein thrombosis

### Comparison of imaging parameters

**Fig. 2:**
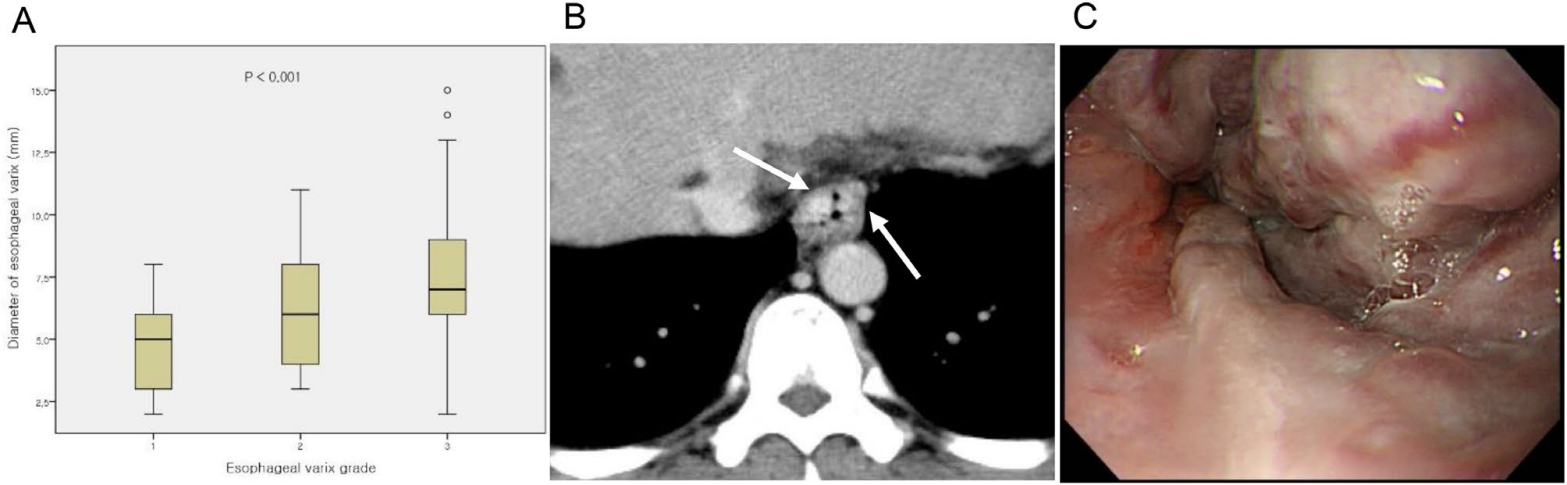
**A** Relationship between CT variceal diameter and endoscopic grading. **B, C** 44-year-old man with liver cirrhosis and large esophageal varices. Portal phase CT image of esophagus shows multiple nodular, enhancing, intraluminally protruding lesions (arrows) within esophagus wall. In this patient, largest varix was measured as 10.7 mm. Endoscopic image shows multiple large varices

Correlation between the grade of EV observed during endoscopy and the diameter of EV on CT was significant (*p* < 0.001) (Fig. 2). The imaging data of both the early and non-early bleeding groups are presented in Table 3. There was no difference of the endoscopic findings. The grade of EV (*p* = 0.238), number of varices (*p* = 0.244), presence of concomitant gastric varices (*p* = 0.807), and number of EVL (*p* = 0.676) were not significantly different between groups. On CT findings, EV diameter was significantly different between groups. The EV diameter was larger in the early than non-early bleeding group (8.42 ± 2.74 vs. 6.84 ± 2.33; *p* = 0.005).

**Table 3:** Comparison of imaging date between the patient groups

Variables	Non-early bleeding (N = 266)	Early bleeding (N = 19)	P value
Endoscopic findings			
Esophageal varices grade			0.238
F1	16 (6.0%)	0 (0%)	
F2	88 (33.1%)	4 (21.1%)	
F3	162 (60.9%)	15 (78.9%)	
Number of varices	3.80 ± 0.49	3.95 ± 0.23	0.244
Extent of esophageal varies			0.804
Fi	48 (18.0%)	3 (15.8%)	
Fm	218 (82.0%)	16 (84.2%)	
Gastric varices	77 (28.9%)	6 (31.6%)	0.807
Number of EVL	3.72 ± 1.40	3.58 ± 2.04	0.676
CT findings			
Esophageal varix diameter (mm)	6.84 ± 2.33	8.42 ± 2.74	0.005
Portal vein diameter (mm)	14.82 ± 2.64	14.22 ± 2.76	0.350

Data are presented as number (%) or mean ± SD.

### Multivariate analysis of risk factors for early bleeding after EVL

**Fig. 3:**
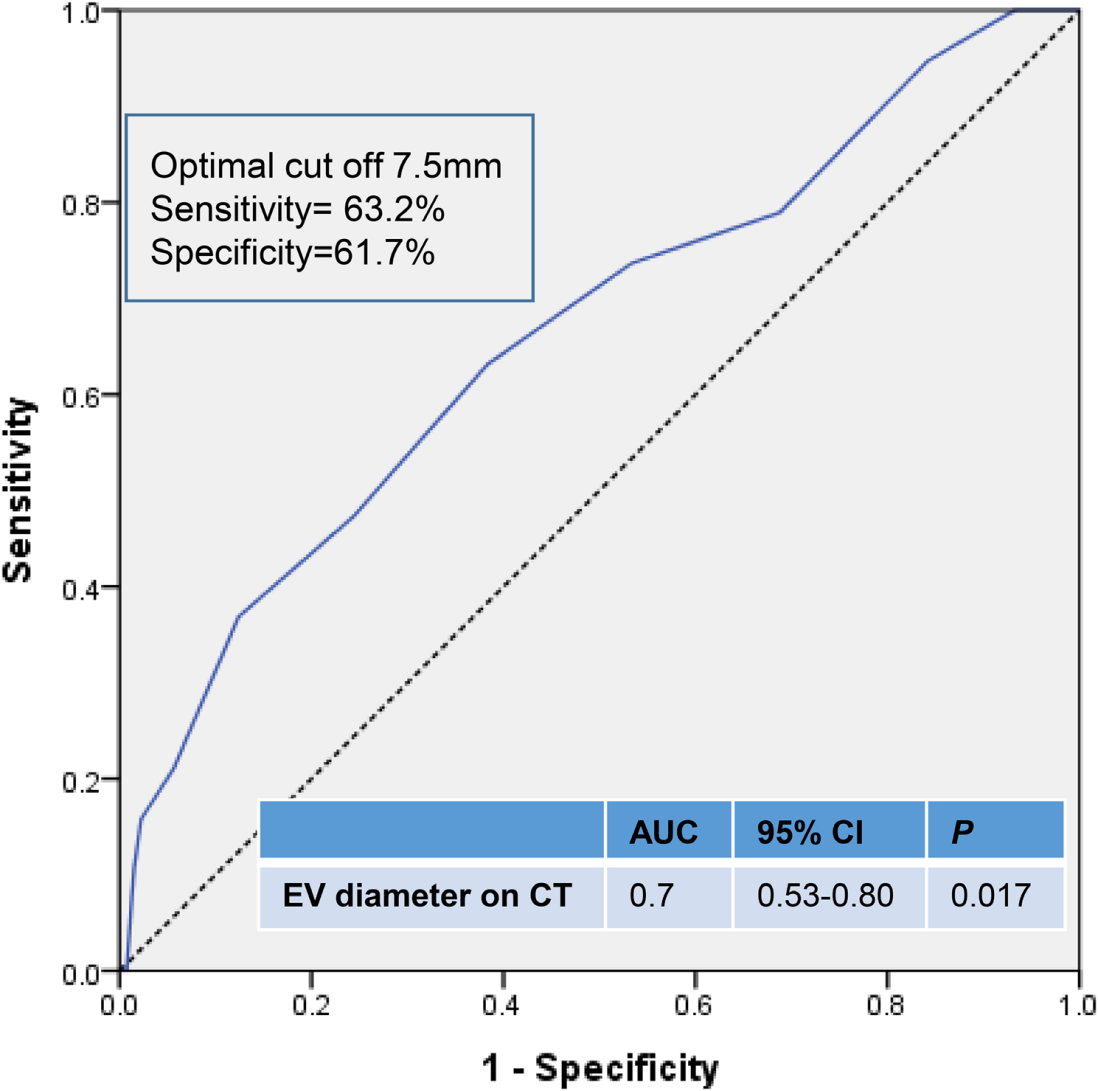
ROC curve shows the predictive ability of esophageal diameter on CT for predicting the early bleeding after EVL.

Four variables (*p* < 0.20) were identified in univariate analysis; total bilirubin, PT, presence of ascites, and EV size. In multivariate analysis, EV diameter on CT was the only independent predictive factor for early bleeding after EVL (OR 1.336, 95% CI 1.099–1.624, *p* = 0.004) (Table 4). We calculated the cut off value, which was 7.5 mm and had a sensitivity and specificity of 63.2% and 61.7% (area under the curve of 0.7), respectively; to determine the optimal EV diameter on CT (Fig. 3).

**Table 4:** Multivariate analysis of risk factors for early bleeding after EVL

Variables	P value	OR	95% CI
Total bilirubin	0.170	1.054	0.977–1.138
Prothrombin time	0.553	1.051	0.893–1.236
Ascites	0.344	1.720	0.560–5.288
Diameter of esophageal varix	0.004	1.336	1.099–1.624

## Discussion

EVL is an effective method to treat or prevent esophageal variceal bleeding. Bleeding after EVL, which was mainly associated with EVL-induced ulcer bleeding, occurs in 2.8 to 7.8% of patients [10–12, 25, 26], and the mortality is reported to be as high as 52% [10]. Previous studies suggested several factors associated with early bleeding after EVL. Poor liver function has been well known as a predictive factor of early bleeding in patients with liver cirrhosis. Yang et al. demonstrated that end-stage liver cirrhosis (Child-Pugh C) was an independent risk factor for early bleeding within 14 days of EVL [27]. Berreta et al. reported that Child-Pugh C classification was an independent predictive factor for rebleeding-related death [28]. Other studies found that each variable for Child-Pugh classification was an independent risk factor for early bleeding or rebleeding within 4 weeks of EVL. PT or index, albumin, presence or severity for ascites, and MELD were reported as independent predictive factors for early bleeding or rebleeding after EVL [10–13, 26].

Regarding the endoscopic findings of varices, previous studies suggested several risk factors. Xu et al. reported that varices throughout the whole extent of the esophagus are more dangerous than those that are limited to the middle and lower esophagus for early rebleeding [11]. Cho et al. reported that concomitant F3 GV was a risk factor for EVL-induced ulcer bleeding [12]. The risk factors not only related to severity of liver function, but also related to EVL procedure. Lee et al. found that the higher number of EV ligations was significantly associated with rebleeding within 6 weeks of EVL due to extensive surface area of the mucosal injury [29]. Xu et al. also proved that the more rubber bands that were used for ligation, the greater the possibility of rebleeding within 2 weeks [11]. Sinclair et al. found that a higher band-induced ulcer bleeding rate within 4 weeks were observed following EVL for acute variceal hemorrhage, which EVL is unavoidable than prophylactic EVL [26]. In our study, we collected data previously reported as risk factors and evaluated whether they were useful as predictors for early bleeding, however, we could not draw convincing conclusions. This can be explained by sampling differences, heterogenous populations, different definitions such as early bleeding and bleeding period, and selection bias due to retrospective study design.

Considering the morbidity and mortality associated with variceal hemorrhage in patients with cirrhosis, it is recommended that these patients should undergo endoscopic screening for the evaluation of gastroesophageal varices [4]. However, it has been observed that there are costs and risks associated with endoscopy and endoscopy, which can be poorly tolerated, particularly in patients with cirrhosis [30–32]. Patients with cirrhosis sometimes undergo CT scans to screen for HCC when ultrasound surveillance is suboptimal [33, 34]. With the recent advancement in multiphase CT, the evaluation of portosystemic collateral vessels using CT scan is possible. Previous studies have evaluated the correlation for varices between CT and endoscopy findings and have reported that CT results were comparable to those of endoscopy in detecting varices. Furthermore, agreement between radiologists, regarding the size of varices, was better than between endoscopists [14–16]. In a prospective study, CT was found to have approximately 90% sensitivity and 50% specificity in detecting esophageal varices [14]. A recent retrospective study reported a CT sensitivity of 98.96%, specificity of 100%, and diagnostic accuracy of 98.97% for EV in patients with chronic liver disease [35]. Therefore, CT can be considered as a potential noninvasive, less expensive and more compliant EV detection method compared to the invasive endoscopic screening modality.

To date, previous studies investigated the diagnostic accuracy and correlation between CT and endoscopic findings, these studies have not evaluated CT findings associated with variceal bleeding. There was only one study which evaluated the CT findings to predict variceal hemorrhage, but no studies have explored the prognostic value of CT in predicting the risk of early bleeding after EVL. To our knowledge, our study is the first to investigate the associations between CT findings and early bleeding after EVL in patients with cirrhosis. Results from our study show that a larger EV diameter on CT is the only independent predictive factor for early EV bleeding. Differences in distension of the esophagus, which cause some of the discrepancy in accessing variceal size during endoscopy and CT, may account for our results. While the esophagus is insufflated or deflated during endoscopy, the transluminal pressure and, consequently, EV size are variable. When the esophagus is nondistended during CT, the hemodynamics of the varices are maintained. Therefore, the assessment of varices in a nondistended state during CT may allow for a more accurate measurement of varix diameter.

There are several limitations of our study. First, this was a retrospective study with selection bias and the number of patients who experienced early bleeding were relatively few. Second, early bleeding events were not all confirmed by endoscopy and therefore, we could not classify bleeding events as EVL-induced ulcer bleeding or new variceal bleeding. Third, although we confined time interval between endoscopy and CT (≤ 60 days), interval progression or regression of EV cannot be entirely ruled out. Fourth, imaging measurements had variability in findings. There may be the variability in CT findings between radiologists, as well as in endoscopic findings between endoscopists. Furthermore, in endoscopic findings, there may be inaccuracy for assessing the grade of EV during the acute variceal bleeding. Lastly, although we suggest the specific cut-off value of diameter of EV diameter on CT, we could not perform further validation.

In conclusion, a larger EV diameter on CT is a risk factor for early bleeding after EVL treatment. The optimal cut-off value for EV size remains to be determined. Therefore, further large cohort, prospective design are required to determine the optimal cut-off value and role of CT as a noninvasive screening tool for the early identification of high-risk groups and change of treatment strategies to improve patient outcomes.

## Data Availability

The datasets generated and/or analysed during the current study are not publicly available due to protect the privacy of the patient, but are available from the corresponding author on reasonable request.
